# Isothiocyanates as Tubulin Polymerization Inhibitors—Synthesis and Structure–Activity Relationship Studies

**DOI:** 10.3390/ijms241813674

**Published:** 2023-09-05

**Authors:** Renata Grzywa, Mateusz Psurski, Anna Gajda, Tadeusz Gajda, Łukasz Janczewski

**Affiliations:** 1 Department of Organic and Medicinal Chemistry, Faculty of Chemistry, Wrocław University of Science and Technology, Wybrzeże Wyspiańskiego 27, 50-370 Wrocław, Poland; renata.grzywa@pwr.edu.pl; 2 Department of Experimental Oncology, Hirszfeld Institute of Immunology and Experimental Therapy, Polish Academy of Sciences, 12 Rudolf Weigl St., 53-114 Wrocław, Poland; mateusz.psurski@hirszfeld.pl; 3 Institute of Organic Chemistry, Faculty of Chemistry, Lodz University of Technology, 116 Stefan Żeromski St., 90-924 Łódź, Poland; anna.gajda@p.lodz.pl (A.G.); tadeusz.gajda@p.lodz.pl (T.G.)

**Keywords:** isothiocyanates, microtubules, anticancer activity, tubulin polymerization inhibitors, α tubulin, β tubulin, sulforaphane, phosphorus analog of sulforaphane, benzyl isothiocyanate, molecular docking

## Abstract

Among the various substances that interfere with the microtubule formation process, isothiocyanates (ITCs) are the group of compounds for which the binding mode and mechanism of action have not yet been explained. To better understand the structure–activity relationship of tubulin-isothiocyanate interactions, we designed and synthesized a series of sixteen known and novel, structurally diverse ITCs, including amino acid ester-derived isothiocyanates, bis-isothiocyanates, analogs of benzyl isothiocyanate, and phosphorus analogs of sulforaphane. All synthesized compounds and selected natural isothiocyanates (BITC, PEITC, AITC, and SFN) were tested in vitro to evaluate their antiproliferative activity, tubulin polymerization inhibition potential, and influence on cell cycle progression. The antiproliferative activity of most of the newly tested compounds exceeded the action of natural isothiocyanates, with four structures being more potent as tubulin polymerization inhibitors than BITC. As a confirmation of anti-tubulin activity, the correlation between polymerization inhibition and cell cycle arrest in the G_2_/M phase was observed for the most active compounds. In light of the biological results indicating significant differences in the impact of structurally diverse isothiocyanate on tubulin polymerization, in silico analysis was conducted to analyze the possible mode of isothiocyanate-tubulin binding and to show how it can influence the polymerization reaction.

## 1. Introduction

**Figure 1:**
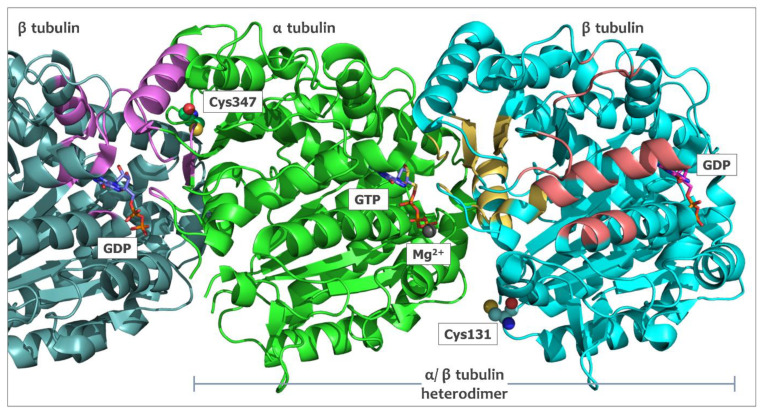
The arrangement of surface-accessible cysteine residues, α tubulin Cys347 and β tubulin Cys131 (sphere representation), in the structure of the α/β tubulin heterodimer. The dimer was extracted from the microtubule polymer structure (7sj7.pdb) together with the preceding β tubulin unit. GTP+Mg^2+^ bound with α tubulin subunit and GDP located in the pocket on the surface of β tubulin subunits are shown in the stick representation. The vinca binding site is shown in violet, the pocket that interacts with colchicine in yellow, and the taxol binding site in salmon. Shown binding residues for these cavities were selected on the basis of the distance (7 Å) from the ligand; 1z2b.pdb for the vinca and the colchicine domains and 1jff.pdb for the taxol binding site.

Microtubules play a key role in cell proliferation, trafficking, signaling, and migration. They are dynamic polymers composed of α and β tubulin arranged during the polymerization process into a cylindrical tubular form that can be many micrometers long. In the context of cancer cells, their role during mitotic division as highly dynamic mitotic spindle constituents is especially important. Compounds that alter microtubule functionality have been proven to be highly potent anticancer drugs, and there is a continuous interest in the development of novel agents with a mode of action focused on microtubules. Vinca alkaloids (e.g., vincristine, vinblastine, vinflunine) isolated more than 50 years ago, as well as taxanes (e.g., paclitaxel and docetaxel), with over 40 years of history in oncology, are still frequently utilized in various chemotherapy regimes in a broad range of solid tumors and hematological malignancies. In recent years, a large number of chemically diverse substances capable of altering microtubule polymerization were synthesized or isolated from natural sources, including colchicine derivatives, noscapine, ombrabulin, eribulin, and laulimalide, many of which successfully passed the preclinical evaluation and reached the stage of clinical trials [1]. On the basis of the mechanism of action, these antimitotic agents are assigned as polymerization enhancers (like taxanes) or inhibitors (vinca alkaloids, colchicine, and its derivatives). Regarding the tubulin binding site, they can be described as vinca–domain binders (vinca alkaloids, eribulin), colchicine–domain binders (colchicine, 2-methoxyestradiol, indulin) and taxol–domain binders (taxanes and cyclostreptin) (Figure 1) [2,3,4].

Isothiocyanates (**ITCs**), one of the major glucosinolates (a class of sulfur-containing secondary metabolites found abundantly in cruciferous vegetables) breakdown product, are an example of tubulin polymerization inhibitors that do not share a common mechanism of action with any other microtubule–binding agents mentioned above. Since the discovery of the antiproliferative activity of sulforaphane (**SFN**) [5], naturally occurring isothiocyanates have received constant interest as potentially useful anticancer agents. Numerous studies, both in vitro and in vivo, showed their potential as chemopreventive and antitumor agents [6]. In the last 25 years, multiple studies have revealed several different modes of isothiocyanates’ biological activity, the majority of which are associated with the presence of a highly electrophilic carbon atom in the isothiocyanate moiety and its high reactivity toward the sulfhydryl group. Glutathione (an intracellular redox status guardian), due to its abundance, has long been recognized as a primary and major isothiocyanates’ molecular target. However, recent studies indicate that the conjugation with glutathione followed by the mercapturic acid pathway is mainly responsible for the rapid accumulation of isothiocyanates (their intracellular concentrations can exceed 100–200 times the extracellular concentration after 0.5–3 h of exposure [7]). An accompanying drastic decrease in the glutathione intracellular pool is often associated with the elevation of the reactive oxygen species (ROS) level and renders secondary molecular targets susceptible to isothiocyanates activity.

At least 30 proteins were recognized as direct molecular targets for isothiocyanates, most of them associated with cancer initiation, progression, epithelial–mesenchymal transition (EMT) phenomenon, self-renewal and metastasis of cancer stem cells [6,8]. Among them, those associated with mitotic division take a special place. Down-regulation of Cdc25C or induction of cyclin B1 associated with phosphorylation of Cdk1 and modulation of other cyclins’ levels are often an outcome of isothiocyanate treatment that leads to a prolonged cell cycle arrest in G_2_ or M phase, mitotic spindle disruption, and eventually cell death via mitotic catastrophe. Furthermore, these processes are commonly accompanied by competitive inhibition of histone deacetylases, enzymes responsible for epigenetic control of gene expression, as well as thiocarbamoylation of several sulfhydryl groups in α and β tubulin. The interactions with tubulin are especially interesting since they not only lead to tubulin polymerization inhibition but also to substantial structural changes and degradation by the ubiquitin-proteasome mechanism—a unique feature of isothiocyanates never observed neither for ‘standard’ tubulin polymerization modulators nor other thiol-interacting agents such as arsenic trioxide, hydroquinone and 4-hydroxynonenal. Moreover, proteomic analysis identifies α and β tubulin as proteins most frequently modified by certain isothiocyanates; for example, studies on H460, A549, and HeLa cancer cells revealed rapid depletion of all isoforms of α and β tubulin (but not γ tubulin) after treatment with benzyl (**BITC**) or phenethyl (**PEITC**) isothiocyanates [8,9,10,11]. Among modified cellular proteins, different isoforms of tubulin rank as the first four with the highest change exceeding 40% level decrease after 6 and 24 h [11].

Until now, all studies considering isothiocyanates as anti-tubulin agents were conducted using the four most abundantly studied, naturally occurring compounds, namely **SFN**, **BITC**, **PEITC** and allyl isothiocyanate (**AITC**). It largely limits our understanding of the structure–activity relationship and further the tubulin polymerization inhibition mechanism. Such information would be a useful starting point for the re-design and synthesis of isothiocyanates with the chemical structure optimized for tubulin polymerization inhibition. In the presented study, we made the first step toward deciphering the mode of binding and mechanism behind the inhibition of tubulin polymerization by isothiocyanates, by in vitro examining the inhibitory potential of a series of structurally diverse **ITCs** and by comparing the biological results with analysis of in silico ITC–tubulin binding models.

## 2. Results

### 2.1. Chemistry

Despite the relatively small structural diversity of the most common natural isothiocyanates, Zhang and Chung groups [9,12] independently provided some hints regarding the structure–activity relationship underlying their activity as tubulin polymerization inhibitors. Among the compounds tested, **BITC** proved to be the most potent inhibitor. **PEITC** showed slightly lower activity, while **AITC** and **SFN** activity was negligible in this regard [9,12]. To address the problem of limited diversity of the isothiocyanates utilized in tubulin polymerization inhibition studies, we designed a set of structurally diverse compounds **1**–**16**, e.g., representing amino acid esters-derived isothiocyanates, *bis*-isothiocyanates and phosphorus-containing compounds (**P-ITCs**).

**Figure 2:**
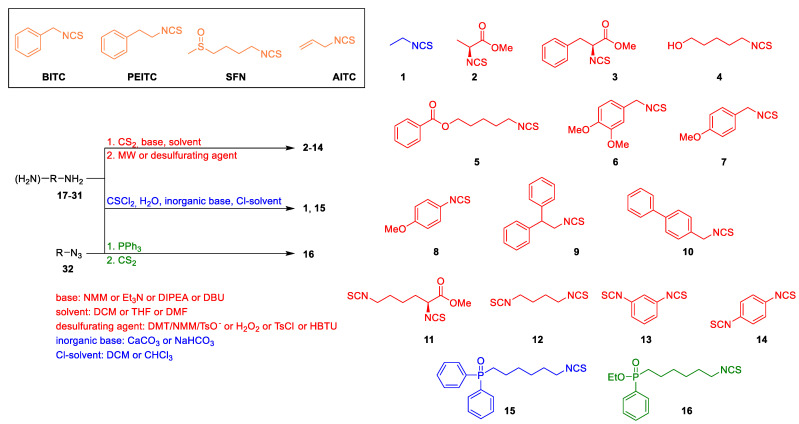
Synthesis and structure of the tested ITCs **1**–**16**. ITCs **1** and **15** were synthesized using thiophosgene; ITCs **2**–**14** were synthesized using a desulfurating agent; ITC **16** was synthesized in tandem with the Staudinger/aza-Wittig reaction.

All tested isothiocyanates **1**–**16** have been synthesized by us, according to Figure 2. Among them, compound **5** has not been previously described in the literature. Detailed synthetic procedures for all compounds (**1**–**16**) [13,14,15,16,17,18,19], together with substrates **17–32** structures, are presented in the Supplementary Materials. The main approach to the synthesis of isothiocyanates from the corresponding amines (or diamines) utilized carbon disulfide (CS_2_) in the presence of an organic base (e.g., Et_3_N) followed by desulfuration of the intermediate dithiocarbamate. The following desulfurating agent was used: the 4-(4,6-dimethoxy-1,3,5-triazin-2-yl)-4-methylmorpholinium toluene-4-sulfonate (DMT/NMM/TsO^─^) for compounds **2**, **3** and **11 [13]**, hydrogen peroxide for compounds **4**–**6 [14]**, and tosyl chloride for compound **7 [15]**. Compounds **8**–**10** were obtained by microwave-assisted (MW) synthesis [16], and diisothiocyanates **12**–**14** were synthesized using HBTU [17]. To obtain 6-(isothiocyanatohexyl)diphenylphosphino oxide (**15**), amine was treated with thiophosgene and NaHCO_3_ [18], while compound **16** was prepared from the corresponding ethyl (6-azidohexyl)(phenyl)phosphinate (**32**) in tandem reaction with CS_2_ and triphenylphosphine (Ph_3_P) [18]. In addition, compound **1** was synthesized from the parent primary amine in reaction with thiophosgene in the presence of aq. CaCO_3_ [19]. All aforementioned compounds were obtained with satisfactory yields (25–98%) and with high purity.

### 2.2. In Vitro Antiproliferative Activity of ITCs

All synthesized ITCs (**1**–**16**) and reference compounds (**BITC**, **PEITC**, **SFN**, and **AITC**) were tested to establish their antiproliferative activity in four cancer cell lines: LoVo (colon adenocarcinoma), A2780 (ovary cancer), MV-4-11 and U-937 (both leukemia) (Table 1). With the exception of **1**, **8** and **13**, all exhibited IC_50_ significantly below 50 µM (the highest compound concentration tested) without explicit cell line specificity, confirming previous numerous observations that the isothiocyanates multimodal mechanism of action minimizes risk of isothiocyanates resistance. Outstanding antiproliferative activity was observed for **6** (IC_50_ [μM]: LoVo = 1.92 ± 0.38; A2780 = 1.38 ± 0.2; MV-4-11 = 0.87 ± 0.45; U-937 = 2.02 ± 0.60) and **7** (IC_50_ [μM]: LoVo = 1.70 ± 0.26; A2780 = 1.24 ± 0.09; MV-4-11 = 0.81 ± 0.2; U-937 = 1.53 ± 0.12) (methoxy-substituted benzyl isothiocyanates in position 3, 4 or 4, respectively) for which activity was almost two times higher compared to parental **BITC**. At the same time, closely related *p*-methoxyphenyl isothiocyanate **8** showed negligible activity, while the replacement of the OMe group with the second isothiocyanate moiety produced **14** with activity comparable to **PEITC**. Interestingly, in the case of aryl diisothiocyanates, the change in the configuration of the isothiocyanate group from *para* (**14**) to *meta* position (**13**) resulted in a dramatic loss of antiproliferative activity; however, aliphatic diisothiocyanate **12** was characterized as the most potent among all compounds tested (IC_50_ [μM]: LoVo = 0.68 ± 0.09; A2780= 0.32 ± 0.07; MV-4-11 = 0.22 ± 0.05; U-937 = 0.72 ± 0.06). Lysine-derived diisothiocyanate (**11**) (IC_50_ [μM]: LoVo = 1.64 ± 0.53; A2780 = 1.00 ± 0.22; MV-4-11 = 1.66 ± 0.22; U-937 = 2.95 ± 1.07), but not isothiocyanate derivatives of alanine and phenylalanine (**2** and **3**, respectively), exhibited activity similar to other highly potent compounds (e.g., **5** and **6**) including most hydrophobic structures in the set—bis-phenyl isothiocyanates **9** and **10**. The presence of a phosphorus atom in **P-ITCs 15** and **16** does not increase the biological activity of the compounds in the cancer cell lines tested (Table 1).

### 2.3. ITCs as Tubulin Polymerization Inhibitors

**Figure 3:**
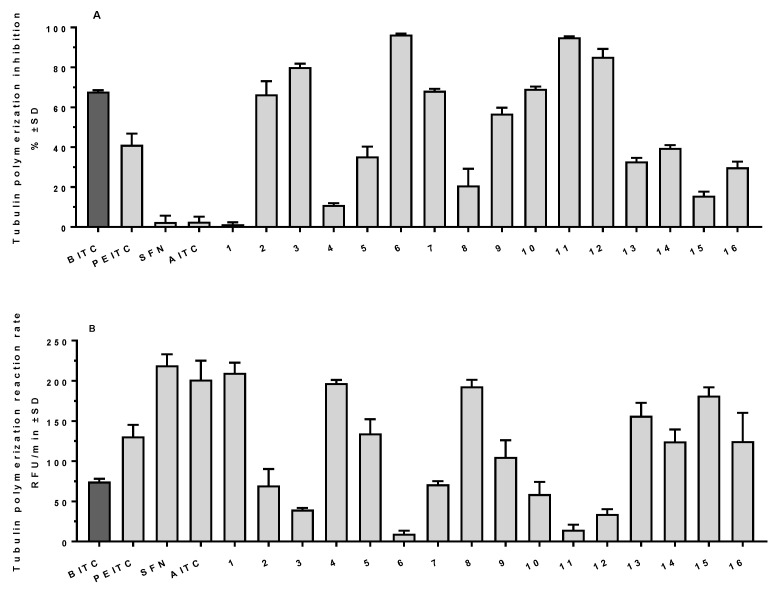
Tubulin polymerization test results for all twenty compounds tested. (**A**) Results presented as tubulin polymerization inhibition calculated on the basis of microtubule mass observed for the steady state. (**B**) Results presented as tubulin polymerization rate during the growth phase.

In further studies, all twenty compounds were tested to assess their activity as polymerization inhibitors in vitro using a cell-free assay. First, all compounds were applied at a 25 µM concentration to discriminate their potential based on the mass of microtubules built up after a polymerization lasting 60 min (steady state) and the reaction rate (growth phase). **BITC** was identified as the most potent inhibitor among the four reference compounds with 67.8% inhibition (determined from microtubule mass) (Figure 3A) and a reaction rate of 74.6 RFU/min (control, vehicle-treated samples showed 220.0 ± 15 RFU/min) (Figure 3B). Structurally similar **PEITC** exhibited significantly lower activity (41.2% and 130.8 RFU/min, respectively), while **SFN** and **AITC** had negligible potential with inhibition not exceeding 3% (Figure 3A) and reaction rate similar to the control. Among the 16 compounds tested, 8 showed activity comparable to **BITC** (namely **2**, **3**, **6**, **7**, **9**–**12**), with 3,4-dimethoxybenzyl isothiocyanate (**6**), (*S*)-methyl 2,6-diisothiocyanatohexanoate (**11**) and 1,4-diisothiocyanato butane (**12**) identified as highly active agents with polymerization inhibition exceeding 85% (Figure 3A), and a reaction rate reduced below 35 RFU/min (Figure 3B) when applied at 25 µM concentration. All compounds shared the same activity pattern—both the reaction rate and final microtubule mass were reduced simultaneously. It should be noted that the same compounds showed activity higher than that of **BITC** in antiproliferative tests. The lowest activity was observed for ethyl isothiocyanate (**1**), 5-hydroxypentyl isothiocyanate (**4**), 4-methoxyphenyl isothiocyanate (**8**), and 6-(isothiocyanatohexyl)diphenylphosphino oxide (**15**) with an inhibition not exceeding 20% (Figure 3A) and reaction rate above 180 RFU/min (Figure 3B). Again, the list of the least active compounds in the in vitro polymerization assay and in the antiproliferative assay in cellulo coincides. However, it should be pointed out that low tubulin polymerization inhibition does not necessarily indicate low antiproliferative activity (*vide* compound **5** with its high antiproliferative activity, especially on MV-4-11 cell line, and only moderate to low tubulin polymerization inhibition), which is a reflection of general isothiocyanates characteristic, that they have several different intracellular molecular targets, and can exhibit biological activity by influencing diverse biological processes. Additionally, for compounds **8** and **13**, we observed some activity in the tubulin polymerization assay (ca. 20% and 33%, respectively) (Figure 3A), which exceeded the observed activity. For example, for **SFN**, these compounds still had negligible antiproliferative activity, indicating that other processes (plausibly their ability to cross the cell membrane and reach intracellular targets) impair their potential to be potent inhibitors (Figure 3 and Table 1).

**Figure 4:**
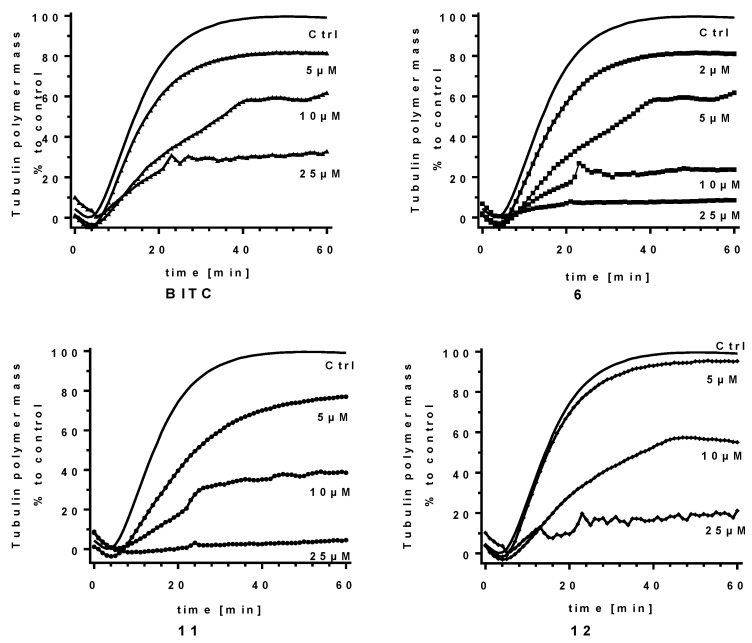
Tubulin polymerization curves acquired for vehicle-treated control (Ctrl) and compounds applied at indicated concentrations. Curves are based on average values obtained from two independent repeats performed for each compound and concentration.

Three of the most active compounds (namely **6**, **11** and **12**) together with **BITC** were further tested using various concentrations of compounds to determine the dose–response relationship (Figure 4). A clear dose–response relationship was observed for all four compounds, with **6** identified as the most active with IC_50_ (compound concentration that reduced the mass of the polymer observed in steady state by 50% compared to the control) value of 6.6 µM (8.2 µM for **11**, 11.4 µM for **12** and 13.0 µM for **BITC**). It should be noted that 3,4-dimethoxybenzyl isothiocyanate (**6**) applied at 2 µM was equally active as closely related **BITC** applied at 5 µM (Figure 4).

### 2.4. ITCs Influence on Cell Cycle Progression on MV-4-11 Cells

**Figure 5:**
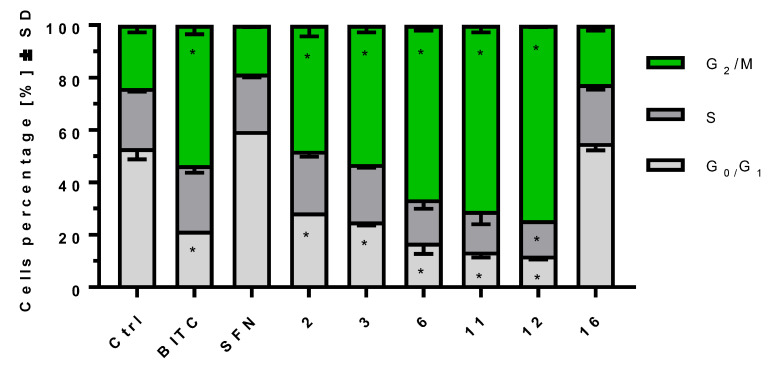
Cell cycle phases profile for MV-4-11 cells treated for 24 h. with various compounds at 5 µM. *—*p* < 0.05, one-way ANOVA with Dunnett’s post-hoc test for multiple comparisons (with the corresponding cell cycle phase in the Ctrl sample treated as a reference).

Microtubule polymerization inhibition is often correlated with cell cycle arrest in the G_2_/M phase. Selected compounds were tested for their potential as cell cycle arresters in order to check if there is a correlation between the compounds’ activity as tubulin polymerization inhibitors and their ability to impair the cell cycle. The results obtained on MV-4-11 cell line after 24 h. of treatment with four most potent tubulin polymerization inhibitors and two less active compounds (namely **SFN** and **16**) used at 5 µM concentration indicated a positive correlation between tubulin polymerization inhibition and cell cycle arrest induced by the compounds. Compounds **6**, **11** and **12** increased the G_2_/M cells percentage from 24.0% ± 2 observed for control cells to 66.5% ± 2, 71.0% ± 2 and 74.5 % ± 0.5, respectively. At the same time benzyl isothiocyanate (**BITC**), as well as compounds **2** and **3**, which exhibited moderate tubulin polymerization inhibition, caused moderate cell cycle arrest with G_2_/M cell percentages of 53.5% ± 2, 48.0% ± 3 and 53.5% ± 1.5, respectively. The least potent tubulin polymerization inhibitors (**SFN** and **16**) also proved to be the least potent cell cycle arresters with G_2_/M cell percentages of 19.0% ± 1 and 22.5% ± 0.5, respectively (Figure 5).

The combined results of the biological studies showed that the chemical structure of isothiocyanates strongly influences their potential as tubulin polymerization inhibitors and that there is a significant correlation between tubulin polymerization inhibition and cell cycle arrest. However, strong tubulin polymerization inhibition properties are not necessary for isothiocyanates to reflect high antiproliferative activity, yet in those cases, their mechanism of action engages different intracellular targets (as indicated by the results of the cell cycle assay).

### 2.5. In Silico Studies for Isothiocyanates–Tubulin Interactions

To better understand the basics of structure–activity relationship observed for tested compounds, we decided to analyze the ITC–tubulin binding in silico. The reversible and highly dynamic process of microtubule filament formation assembles subunits of α and β tubulin heterodimers in a GTP-dependent manner. Binding sites for multiple compounds with anticancer properties were already identified for both α and β isoforms [20], including cysteine residues covalently modified by isothiocyanates. The identified, tubulin-derived, ITC-bound peptides indicate a modification of Cys127, Cys347, Cys376 of α tubulin, and Cys12, Cys 239, Cys303, Cys354 of β tubulin [9,12,21], however only α tubulin Cys347 was shown to be modified in cellulo [9]. An analysis of α-1B/β-3 tubulin heterodimer (PDB access no. 6s8l) revealed that among a few surface-accessible cysteine residues, only α tubulin Cys347 and β tubulin Cys131 have a sulfhydryl group facing the heterodimer surface. Cys131 of β tubulin is placed close to the interface between α and β subunits of the heterodimer, while Cys347 is at the opposite site. The electron microscopy structure (PDB access no. 7sj7) shows that this residue is placed on the polymerization surface where the α subunit of the incoming heterodimer interacts with the last subunit β of microtubule (Figure 1). This region of the last β subunit includes the GTP binding site, which, at the time of polymer elongation, is facing the incoming α tubulin Glu254, which is responsible for GTPase activity [22].

The localization of α tubulin Cys347 on the surface directly connecting tubulin units during polymerization suggests that the modification of this residue may influence both the efficiency of the polymerization process and the stability of created structures. Analysis of available α/β tubulin structures, together with data showing that the Cys347 residue is modified by isothiocyanates in cells [9], led to the selection of this position as the main target for molecular docking studies. As a receptor molecule, the structure of the human recombinant α/β tubulin heterodimer was selected (PDB access no. 6s8l). The ligand structures were optimized with the MMS force field [23], and docking studies were performed with the Protein–Ligand ANT System (PLANTS, v. 1.2) [24,25,26]. The docking parameters were set with the binding site center coordinates at the sulfur atom of α tubulin Cys347 and the biding site radius of 10 Å. The only constraint used was the distance between the sulfur of Cys347 and the highly electrophilic carbon of the isothiocyanate group of the ligand. In the case of compound **11**, where the isothiocyanate groups are not symmetrical, both orientations were tested.

**Figure 6:**
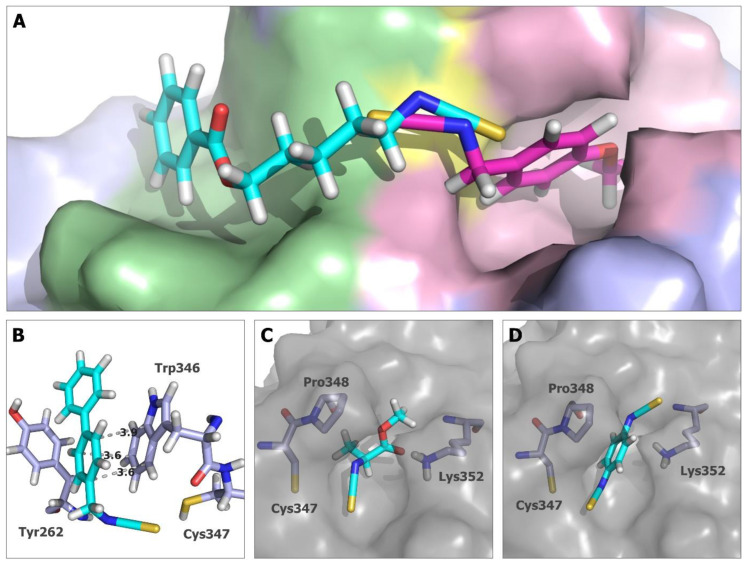
(**A**) Two cavities were identified in the docking study as potential binding pockets for ITCs. The larger cavity, shown in green, was occupied by the best docking poses of larger compounds represented by **5** (shown in cyan). For all remaining tested **ITCs** (represented by compound **7**, in magenta), the best scoring poses were located in the smaller pocket presented in pink. Cys347 is shown in yellow. (**B**) Model of compound **10** bound in the larger cavity built mainly by residues Trp346 and Tyr262. Docking poses of compound **2** (**C**) and compound **14** (**D**) in a small cavity limited by residues Pro348 to Lys352.

The best-scoring docking poses of the analyzed **ITCs** can generally be divided into two groups depending on the binding cavity occupied by the ligand. All smaller compounds, with aromatic and aliphatic side chains, bind to a small narrow pocket located next to Cys347. The pocket size is limited by the polypeptide chain fragment created by Pro348 to Lys352. For larger structures, such as those represented by compounds **5**, **9**, **10**, **15**, and **16**, the best scoring poses were not fitted in this pocket but docked at the opposite site (Figure 6A).

In the case of compounds **5**, **9**, **10**, **15**, and **16**, the scoring results calculated for the best docking poses are, in general, better than the results for compounds that bind in the smaller cavity (see Supplementary Table S1). These results do not reflect the potency of action of these compounds tested in the tubulin polymerization inhibition assay, since the activities of compounds **5**, **15**, and **16** were less than 50% of the best compound and the most active among them (compounds **9** and **10**) had the lowest docking scores. The high-scoring results of these compounds could be partially the consequence of their larger size, but in the case of compounds **5**, **10**, **15**, and **16,** the phenyl ring is located in the cavity built mainly by Tyr262 and Trp346, creating the surface enabling the formation of hydrophobic, including–, stacking interactions (Figure 6B), which can influence the scoring. Compound **9** is not long enough to reach this site (Table S1). A comparison of the biological activity and docking studies for this set of compounds suggests that if the proposed binding mode occurs, the interaction with this cavity does not strongly influence the microtubule polymerization process.

**Figure 7:**
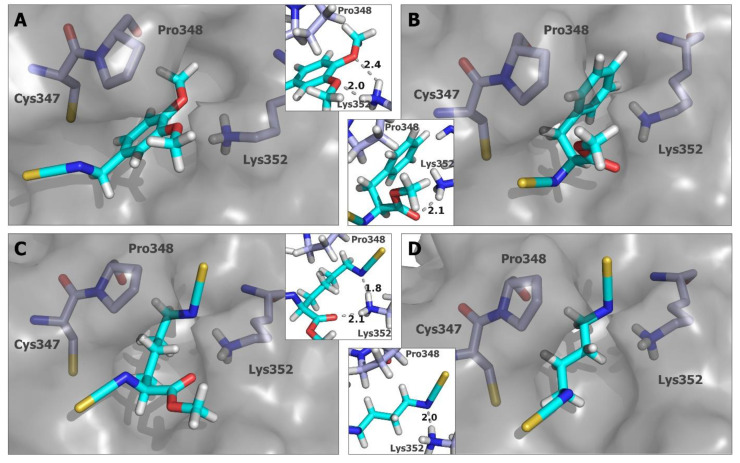
Best docking poses for protein–ligand binding of compounds **6** (**A**), **3** (**B**), **11** (**C**) and **12** (**D**) in a smaller cavity next to Cys347 of α tubulin. The small graphics show the hydrogen bonds between each ligand and Lys352, observed for the docking models (distances in Å).

For all other compounds, the best binding poses occupied the small cavity, and in this case, the docking scores correspond much better to the microtubule polymerization inhibition results. The smallest compounds, **AITC**, **1** (Table S1), and **2** (Figure 6C), were characterized by the worst scoring values, with −45.472 for compound **1** and −50.1264 for **AITC**, and both were inactive in the polymerization inhibition test. The alanine derivative (**2**) has shown better results in docking studies (−56.5898) and is one of the most active inhibitors of tubulin polymerization. The docking pose of **2** shows that a hydrogen bond between the carbonyl oxygen of the ligand and the ε-amino group of Lys352 can be created. Among the **ITCs** bearing the aromatic ring, compounds **8**, **13**, and **14** showed average results in docking studies, as well as in the tubulin polymerization inhibition test. In this case, the presence of the second isothiocyanate group does not greatly improve both the docking results and the inhibition activity. The mode of binding indicates that even though the additional isothiocyanate group (compounds **13**, and **14**—Figure 6D) or the methoxy substituent (compound **8**) are within the distance between the Lys352 to be involved in hydrogen bonds, the structure can be, in fact, too rigid to effectively create a covalent bond with Cys347, and at the same time, hydrogen interactions with the ε-amino group of Lys352. Pro348 residue can especially create a steric hindrance that, in the case of structures with limited rotatable bonds, might reduce the efficiency of ligand–protein binding. Better results were obtained for compounds **6** (Figure 7A) and **7** (Figure 6A), both in vitro and in silico. Docking poses of these structures show that the orientation of the methoxy moiety of the aromatic ring allows the creation of the hydrogen bond with ε-amino group of Lys352 (Figure 7A). When comparing the biological and docking results obtained for compounds **13** and **14** versus compounds **6** and **7**, it becomes apparent that the additional methylene group that separates the aromatic ring from the isothiocyanate group and therefore increases the flexibility of the molecules is crucial for the activity of the compounds.

One of the most active structures when considering tubulin polymerization inhibition is compound **3**—analog of phenylalanine. Interestingly, the best binding model for this **ITC** was characterized by the highest score among compounds bearing an aromatic ring and binding in the small cavity next to Cys347. As in the case of compound **2**, the most distinguishing feature of this model is the formation of a hydrogen bond between the methyl ester group carbonyl oxygen of the ligand and the ε-amino group of Lys352 (Figure 7B). The best-scoring binding model among the compounds with the docking poses located in the cavity created by α tubulin residues from Pro348 to Lys352 was obtained for compound **11** (docking score −68.4147). Interestingly, the compound was also the most active in the tubulin polymerization inhibition test. The docking studies were performed with the ligand (carbon of isothiocyanate group)–receptor (sulfur of Cys347) distance restriction set up as two separate calculations for both isothiocyanate moieties of **11**. The best scoring pose was obtained with the orientation analogs to those presented for models of compounds **2** and **3** with the carbonyl oxygen of the ester group in close proximity with the ε-amino group of Lys352, which allows the creation of the hydrogen bond (Figure 7C). However, in this case, the second isothiocyanate nitrogen is positioned in such a way that the ε-amino group of Lys352 can be involved in the second hydrogen bond. This model once more indicates that interactions with the Lys352 side chain amino group can be important for the binding of **ITC** with Cys347 and can also be translated into the inhibitory potency of microtubule synthesis.

There were a few compounds for which the docking results do not reflect the tubulin polymerization inhibition data. Among them is **PEITC,** which was less active in biological tests than **BITC** but had higher docking scoring results (Table S1). In addition, in the case of some aliphatic compounds (**SFN**, **4**, and **12**), the docking results do not perfectly reflect the inhibition test data. Although compound **12** (Figure 7D) has the docking score of −60.8435, and this result locates it within the range of values obtained for the four most active structures compared to tubulin polymerization inhibition studies, the other two compounds (**SFN** and **4**) were not very active in biological tests, but the docking scores were similar (**SFN**) or even better (**4**) when compared to compound **12**. For those structures (**SFN** and **4**), the binding poses obtained in the docking studies seem somehow squished inside the small cavity; therefore, the obtained mode of binding may not be optimal from the kinetic point of view.

## 3. Discussion

α and β tubulins, as the tubulin polymerization process and the biological processes associated with it, have received a good deal of attention in the past. Mi et al. [9] identified tubulin as one of the isothiocyanates intracellular targets with a direct, covalent modification indicated as a mechanism of action. Fourteen of the twenty cysteines were indicated as plausible targets for covalent modification, but the number of modified cysteines was strongly dependent on the structure of the compounds. **BITC** was identified as the strongest modifier, with even 12 cysteines modified. At the same time, **PEITC** modified nine moieties, and **SFN** only four. These were confirmed in the tubulin polymerization assay, with the conclusion similar to our recent studies that the chemical structure of isothiocyanates strongly influences their ability to alter the tubulin polymerization process, and the efficacy is dependent on the tubulin-ITC ratio. Similar structure–activity relationships were also observed when cytochrome P450 enzyme inhibition by isothiocyanates was tested [27]. This clearly indicates that despite the high chemical reactivity of the isothiocyanate moiety, its biological potential results from sophisticated multi-targeted interactions, not only from the chemical harshness of **ITCs**. Moreover, studies of structure–activity relationships demonstrated that the presence of **ITC** moiety does not guarantee high antiproliferative activity. Compounds **1**, **8** or **13** of the presented study showed negligible activity, but plausibly for different reasons. 

Ethyl isothiocyanate (**1**) appears to be structurally too simple for effective interactions with biological structures, while **8** and **13** suffered from a lack of flexibility and deactivation caused by the phenyl ring directly attached to the nitrogen atom of **ITC**. The same phenomenon was previously observed for phenyl isothiocyanates [28]. The opposite isothiocyanate moieties of **14** positively influenced the antiproliferative activity of compounds—such a *para*-*bis* isothiocyanate could be a good protein cross-linking agent similar to **11** and **12**. Because isothiocyanates are characterized by a multi-targeted mode of action [29], structurally diverse compounds are expected to be differentially active as tubulin polymerization inhibitors with relatively comparable overall biological activity exhibited by them. Compounds such as **SFN**, **5** or **14** were poor tubulin polymerization inhibitors but showed at least moderate antiproliferative activity. The observation is consistent with previous results [8,9], where all three major naturally occurring isothiocyanates (namely **SFN**, **BITC**, and **PEITC**) were identified as comparably potent cell growth inhibitors, nor sharing the same affinity to tubulin (same rule plausibly applies to other targets too). Furthermore, the previously reported positive correlation between the potential of isothiocyanates **BITC**, **PEITC**, and **SFN** as tubulin polymerization and cell cycle arrest in the G_2_/M phase [9] was reflected in our studies with the use of a larger, structurally diverse set of compounds. A significantly higher **SFN** concentration was necessary for substantial cell cycle arrest, and it corresponded directly to the studies focused on tubulin (tubulin polymerization inhibition and tubulin fluorescence microscopy).

The affinity of **ITCs** to α and β tubulin is intriguing since most of the studies clearly indicate a unique mode of action exhibited by **ITCs**. Unlike classic antimitotic agents that bind to specific binding pockets, impairing the polymerization process and (more importantly) influencing the dynamic stability of microtubules, isothiocyanates cause tubulin unfolding, which creates a large, insoluble fraction of those proteins in the cells [10]. This unfolding results from isothiocyanates covalent modification of cysteines (which can reach even 12 modified residues, as mentioned earlier) has to be a gradual process since most cysteines are hidden within the protein structure with surrounding pockets accessible only to small molecules. Since being a small isothiocyanate (like **AITC** or **1**) is not enough to be a potent tubulin polymerization inhibitor, we hypothesize that there must be a single tubulin residue whose modification is crucial for isothiocyanate activity. Moreover, we hypothesize that additional interactions with the surroundings of such residues should substantially influence tubulin biological function. Xiao et al. [10], using a proteomic approach, identified Cys347 as a plausible residue that meets the expectations mentioned above, but without further studies, also noted that a proper understanding of the importance of Cys347 was missing.

The molecular docking results give an insight into the possible mode of binding of **ITCs** with α tubulin Cys347, even if non-covalent docking models do not recreate the final covalent complex formed between Cys347 and the isothiocyanate group. The primary observation suggests the existence of two cavities that can bind isothiocyanate side chains, and the preference toward one of them seems to be determined mainly by the ligand size. For many groups of ligands/inhibitors, more than one binding cavity seems unlikely, but electron-deficient carbon of the isothiocyanate moiety, susceptible to the nucleophilic attack of cysteine thiol, makes the reaction nonspecific and spontaneous. The wide range of structural and size differences between biologically active **ITCs**, including those presented here results of tubulin proliferation inhibition, indicate that in the case of these compounds, their potency of action is difficult to explain by simple structure–activity relationship analysis deduced from interactions with the single and well-defined binding site. In the presented study, the partition of analyzed compounds into groups based on different binding cavities occupied by docking models does not fully coincide with the biological results; however, it strongly indicates that the ability to bind in the pocket built by residues from Pro348 to Lys352 can be connected with higher tubulin polymerization inhibition potential. The second observation concerns the ligand–protein interactions. Most distinctive is the presence of Lys352 in the smaller binding pocket, which can become a hydrogen bond donor. Among the several structures analyzed with additional functional groups that can be a source of a hydrogen bond acceptor, high binding scores were obtained from docking studies for models where the hydrogen bonds with an ε-amino group of Lys352 were observed. These **ITCs** were also among the most active compounds in the polymerization inhibition tests. 

The best examples are structurally diverse compounds **2**, **3** (Figure 6C and Figure 7B, respectively), and **11** with a common feature, methyl ester moiety. Among them, the alanine derivative (compound **2**) showed great improvement in its inhibitory activity when compared with **AITC** and compound **1**—the smallest compounds of the tested set. Moreover, the docking models of compounds **11** and **6**, the two most active **ITCs** in the tubulin polymerization test, created two hydrogen bonds with the Lys352 side chain (Figure 7A,C). 

The analysis of docking models binding in the pocket built by residues Pro348-Lys352 indicates that, in addition to size limitation, a ligand structure cannot be too rigid, the hydrogen bond acceptor is a desired feature and should not be too far from the isothiocyanate group. In addition to these general observations, it is important to mention that properly placed aromatic substituents can be a desirable feature of **ITCs** targeting the Cys347. In this case, the docking models for compounds **9** and **10** suggest that the mode of binding may be located outside the pocket created by residues of Pro348-Lys352. Even though the docking poses a lack of hydrogen bonding with Lys352, those compounds showed high inhibitory activity in the tubulin polymerization test, which suggests that other interactions, conformational changes, or even modification of other cysteine residue may be the leading factors influencing the activity of these compounds.

**Figure 8:**
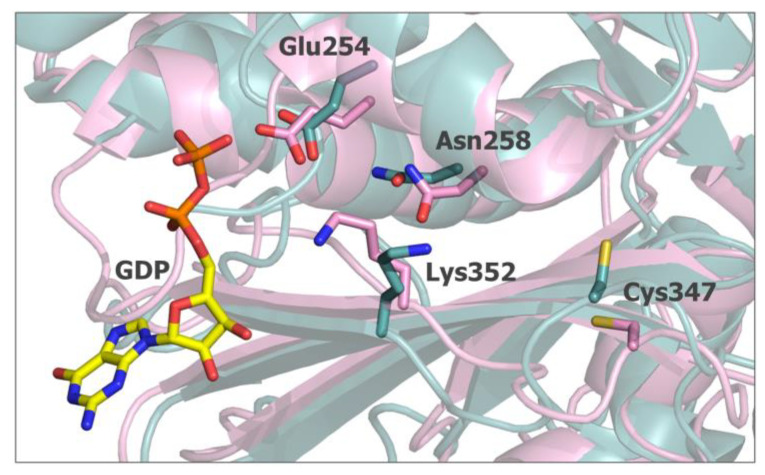
Superposition of α tubulin structures 6s8l.pdb (cyan) and 7sj7.pdb (pink) residues involved in **ITC** binding identified in a docking study (Cys347 and Lys352) and residues of the active site hydrogen bond network described previously [30] shown in a stick representation. The GTP molecule bound to the preceding β tubulin molecule (7sj7.pdb) is included to better visualize the organization of the active site. The receding β tubulin subunits are not shown to maintain clarity.

A close review of the structure of the α/β tubulin heterodimer (6S8L.pdb) compared to the structure of the microtubule polymer (7SJ7.pdb) revealed that the orientation of the Lys352 side chain in the polymer changes with the ε-amino group facing the opposite direction (Figure 8). In such an orientation, this residue is located close to Glu254, which is responsible for the GTPase activity of α tubulin [22]. This change of orientation, the close distance with the catalytic glutamate and the GDP molecule bound within the preceding β tubulin suggest that the residue could be involved in the electrostatic stabilization of GTP during hydrolysis. The involvement of Lys352 in the α tubulin catalytic center formation by the hydrogen bond network between Lys352, Asn258, and Glu254 was previously analyzed by Usui et al. [30]. The authors also showed that the pironet in the inhibitor of tubulin assembly covalently modifies Lys352. The molecular modeling simulations further indicated that the binding of vinca alkaloids on the surface between α tubulin and the preceding β unit takes place with the participation of Lys352 [31].

Based on the data presented here and previous findings [9], we hypothesize that, since α tubulin Cys347 is a target for isothiocyanates (which was confirmed by others in cellulo) [9], the modification of this residue by **ITCs** is responsible for the inhibition of microtubule polymerization. Furthermore, interactions with Lys347 can play a role in this mechanism, and by involving Lys352 via hydrogen bonds, **ITCs** can alter the structure and/or interactions within α tubulin active site and therefore influence the ability to hydrolyze GTP and microtubule filament formation.

In summary, our results provide further insight into the mode of action of isothiocyanates as tubulin polymerization inhibitors. In silico studies, along with the results of biological experiments, are a strong foundation for further rational drug design. Since antimitotic agents are still among the most widely used anticancer drugs, a successful design of another potent tubulin polymerization inhibitor that does not share the mode of action with other agents could be a starting point for clinical studies of such compounds. Furthermore, the apparent importance of Cys347 and the surrounding cavity for tubulin biological functions, further supported by the multi-targeted mode of action of **ITCs**, significantly decreases the risk of drug resistance.

## 4. Materials and Methods

### 4.1. Biological Studies

LoVo, MV-4-11 and U-937 cancer cell lines were purchased from the American Type Culture Collection (ATCC Rockville, MD, USA), and A2780 was purchased from the European Collection of Authenticated Cell Cultures (ECACC; Salisbury, UK). The cell lines were maintained at the Hirszfeld Institute of Immunology and Experimental Therapy (HIIET) and tested for mycoplasma contamination using VenorGeM Classic (Minerva Biolabs, Berlin, Germany), with negative results in all cases. The LoVo cell line was cultured in 1:1 (*v*/*v*) mixture of RPMI-1640 and Opti-MEM (both HIIET, Wroclaw, Poland) supplemented with 5% (*v*/*v*) fetal bovine serum (FBS, GE Healthcare HyClone, Logan, UT, USA), 2 mM L-glutamine and 1 mM sodium pyruvate (all Sigma Aldrich, Poznan, Poland). The A2780, MV-4-11 and U-937 cell lines were cultured in RPMI- 1640 medium w/GlutaMAX^®^ (Thermo Fisher Scientific, Warsaw, Poland) supplemented with 10% (*v*/*v*) FBS. The MV-4-11 and U-937 culture medium were additionally supplemented with 1 mM sodium pyruvate (Sigma-Aldrich, Poznań, Poland). All culture media contained antibiotics: 100 U/mL penicillin and 100 mg/mL streptomycin (both Polfa-Tarchomin, Warsaw, Poland). All cell lines were cultured during all experiments in a humid atmosphere at 37 °C and 5% CO_2_ and passaged twice a week using EDTA-Trypsin (pH 8; HIIET, Wroclaw, Poland) solution as a detachment agent (adherent cell lines only).

### 4.2. In Cellulo Antiproliferative Studies

At 24 h after seeding, the cells in 96-well plates (Sarstedt, Nümbrecht, Germany; density 10^5^ cells/per, 100 µL/well), the tested compounds at concentrations ranging from 30 to 0.1 μM were added (50 mM compounds stock solutions in DMSO were used for serial dilutions). After an additional 72 h, the plates were subjected to the SRB assay (according to a previously described protocol [32] with minor modifications [33], adherent cells) or the MTT assay (according to a previously described protocol [34] with minor modifications [33], non-adherent cells), and the absorbances at 540 nm and 570 nm, respectively, were recorded using a Biotek Hybrid H4 Reader (Biotek Instruments, Bad Friedrichshall, Germany). Compounds at each concentration were tested in triplicate in a single experiment, and each experiment was repeated at least three times independently. The results are presented as the mean cell proliferation inhibition or IC_50_ (half-maximum inhibitory concentration) ± standard deviation (SD), which was calculated using GraphPad Prism 7.05.

### 4.3. Cell-Free Tubulin Polymerization Assay

A ready-to-use tubulin polymerization assay provided by Cytoskeleton, Inc. (Denver, USA) was used (#BK011P) and optimized for inhibition detection. Briefly, 5 µL of 10-times concentrated compound solutions (in relation to their final concentration in a test well) were pipetted onto pre-warmed to 37 °C black, 96-well half-area plates (Greiner Bio-One Gmbh, Leipzig, Germany). Next, the reaction buffer (45 µL/well) containing 1 mg/mL porcine tubulin, 1 mM GTP, 20% (*v*/*v*) glycerol, 80 mM PIPES, pH 6.9, 0.5 mM EGTA, 2.0 mM MgCl_2_ was added, and the fluorescence (excitation = 340 nm, emission = 450 nm) was continuously recorded for 60 min using a Biotek Hybrid H4 Reader. Further analyses were performed in GraphPad Prism 7.05. Two independent repeats were performed for each compound/concentration.

### 4.4. Cell Cycle Analyses

Performed as previously described [35] with minor modifications [33]. Briefly, MV-4-11 cells were seeded on 24-well plates (Sarstedt, Germany) at a density of 15 × 10^4^/well, cultured overnight and treated with compounds at 5 µM concentration (50 mM stock solution in DMSO diluted in culture medium) for 24 h. Next, the cells were washed with PBS and fixed for at least 24 h in 70% (*v*/*v*) ethanol, and then washed with PBS and incubated for 1 h at 37 °C with 500 µL of 8 mg/mL RNAse (Thermo Scientific, Walthman, MA, USA). Next, 50 µL of 0.5 mg/mL propidium iodide solution in PBS (Sigma-Aldrich, Germany) was added to each sample, and after a 20 min incubation in darkness, the samples were analyzed by flow cytometry using a BD LSRFortessa cytometer (BD Bioscience, San Jose, CA, USA). The obtained results were analyzed using ModFit 3.2 software (Verity Software, Los Angeles, CA, USA). Three independent samples were analyzed for each compound.

### 4.5. In Silico Analysis

The molecular docking studies were performed with the crystal structure of the human recombinant α-1B/β-3 tubulin heterodimer (6s8l.pdb) [30] used as a receptor. Models of the isothiocyanate structures were prepared in ChemBio3D 12.0 and optimized with the MM2 force field [23]. The tubulin heterodimer coordinates were extracted from the PDB file, and the preprocessing of protein and ligands structure models was performed with SPORES 1.28 software [36]. Noncovalent, protein–ligand docking calculations were performed using Protein–Ligand ANT System (PLANTS v. 1.2, Eberhard Karls Universität Tübingen, Tübingen, Germany) [24,25,26]. The cysteine residue selected as the ITC-targeted position was Cys347 of α tubulin and the sulfur atom coordinates of this residue were set as a binding site center with a binding site radius of 10 Å. The protein–ligand distance constraint was defined between the Cys347 sulfur and isothiocyanate group of carbon atoms and was set within the distance range 1.5–3 Å with the added weight of −10. The scoring function used for the calculations was *chemplp*. For comparative analysis of structures of tubulin heterodimer (6s8l.pdb) versus microtubule, the electron microscopy structure of wild-type microtubule from recombinant human tubulin was used (7sj7.pdb) [22].

## Figures and Tables

**Table 1:** In vitro antiproliferative activity of tested ITCs **1**–**16**.

	IC_50_ ± SD [µM] *			
Compd	LoVo	A2780	MV-4-11	U-937
**BITC**	4.09 ± 0.96	3.46 ± 0.58	1.44 ± 0.64	8.47 ± 0.22
**PEITC**	8.22 ± 1.64	6.32 ± 1.16	2.67 ± 0.78	9.13 ± 3.12
**SFN**	7.99 ± 2.30	4.40 ± 1.50	3.51 ± 0.09	9.90 ± 3.74
**AITC**	22.58 ± 6.77	11.41 ± 2.50	7.48 ± 2.31	10.50 ± 0.70
**1**	[14.61] ** ± 5.65	[13.55] ** ± 2.12	[8.03] ** ± 4.32	[5.37] ** ± 2.36
**2**	8.68 ± 1.82	4.27 ± 1.12	6.04 ± 1.80	8.03 ± 0.44
**3**	7.88 ± 0.60	6.46 ± 0.28	2.38 ± 0.68	8.16 ± 0.32
**4**	4.01 ± 1.01	3.39 ± 0.56	1.87 ± 0.27	5.50 ± 0.20
**5**	1.84 ± 0.96	3.26 ± 0.31	0.59 ± 0.07	3.28 ± 1.39
**6**	1.92 ± 0.38	1.38 ± 0.20	0.87 ± 0.45	2.02 ± 0.60
**7**	1.70 ± 0.26	1.24 ± 0.09	0.81 ± 0.20	1.53 ± 0.12
**8**	[14.31] ** ± 1.75	[14.25] ** ± 4.32	[18.43] ** ± 4.52	[15.35] ** ± 8.43
**9**	1.86 ± 0.16	1.53 ± 0.44	1.11 ± 0.36	3.22 ± 1.10
**10**	3.97 ± 0.41	2.57 ± 0.81	1.28 ± 0.17	4.15 ± 1.94
**11**	1.64 ± 0.53	1.00 ± 0.22	1.66 ± 0.22	2.95 ± 1.07
**12**	0.68 ± 0.09	0.32 ± 0.07	0.22 ± 0.05	0.72 ± 0.06
**13**	[24.21] ** ± 8.64	[33.65] ** ± 2.12	[28.03] ** ± 3.72	[25.35] ** ± 5.46
**14**	8.00 ± 0.53	7.73 ± 1.66	4.36 ± 1.19	15.85 ± 2.71
**15**	13.21 ± 1.12	11.20 ± 2.36	8.69 ± 1.18	11.21 ± 3.66
**16**	11.87 ± 2.21	9.97 ± 2.69	8.47 ± 3.21	10.22 ± 4.24

*—assessed using SRB method after 72 h. of drug treatment; **—mean proliferation inhibition at 50 µM concentration.

## Data Availability

Data is contained within the article or Supplementary Material.

## Supplementary Materials

The following supporting information can be downloaded at https://www.mdpi.com/article/10.3390/ijms241813674/s1. Refs. [37,38,39,40,41,42,43] are cited in the Supplementary Materials.
