# Supplementary material for: Isothiocyanates as Tubulin Polymerization Inhibitors—Synthesis and Structure–Activity Relationship Studies
Source: Int J Mol Sci. 2023 Sep 5;24(18):13674. doi: 10.3390/ijms241813674 (PMC10531289; doi:10.3390/ijms241813674)
Supplement: Supplementary file 1 [file ijms-24-13674-s001.zip › ijms-2561823-supplementary.pdf]

Isothiocyanates as Tubulin Polymerization Inhibitors—Synthesis and Structure–Activity Relationship Studies

Renata Grzywa [1], Mateusz Psurski [2], Anna Gajda [3], Tadeusz Gajda [3] and Łukasz Janczewski [3,*]

[1] Department of Organic and Medicinal Chemistry, Faculty of Chemistry, Wrocław University of Science and Technology, Wybrzeże Wyspiańskiego 27, 50-370 Wrocław, Poland; renata.grzywa@pwr.edu.pl
[2] Department of Experimental Oncology, Hirszfeld Institute of Immunology and Experimental Therapy, Polish Academy of Sciences, 12 Rudolf Weigl St., 53-114 Wrocław, Poland; mateusz.psurski@hirszfeld.pl
[3] Institute of Organic Chemistry, Faculty of Chemistry, Lodz University of Technology, 116 Stefan Żeromski St., 90-924 Łódź, Poland; anna.gajda@p.lodz.pl (A.G.); tadeusz.gajda@p.lodz.pl (T.G.)
* Correspondence: lukasz.janczewski@p.lodz.pl; Tel.: +48-(42)631-31-45

Table of contents:

1. General information S2
2. Synthetic procedure for ITCs **1-16** S2
3. Table S1 .... S9
4. References S14

1. General information

NMR spectra were measured on a Bruker AC–TM DRX 300 and Bruker Avance II Plus 700 MHz (Bruker Corporation, Billerica, MA, USA) spectrometer in a $CDCl_3$ solution. Chemical shift (δ) are reported in ppm and coupling constants (J) in Hz. ^1H and ^{13}C NMR spectra were referenced according to the residual peak of the solvent based on literature data. Chemical shift (*δ*) were reported in ppm and coupling constants (*J*) in Hz. ^{13}C NMR spectra were proton-decoupled. A Bruker MicrOTOF-Q II spectrometer (Bruker Daltonics, Germany) equipped with an Apollo II electrospray ionization source with an ion funnel was used for the acquisition of the high-resolution electrospray ionization (ESI) mass spectra. Flash chromatography was performed using a glass column packed with Baker silica gel (30–60 m). For TLC, silica gel with a 254 nm indicator on Al foils (Sigma–Aldrich, St. Louis, MO, USA) was used. All reagents and solvents were purchased from Sigma-Aldrich (Poland) and used as obtained.

2. Synthetic procedure for ITCs 1-16

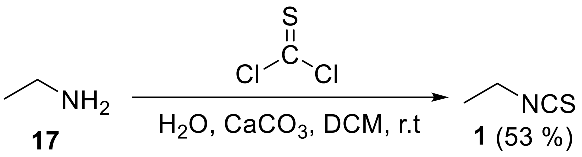

Figure S1. Synthesis of ethyl isothiocyanate (**1**) using thiophosgene.

ITC **1** was synthesized from ethylamine (0.13 mL, 2 mmol) (**17**) in the presence of thiophosgene (0.31 mL, 4 mmol) in a two-phase system ($CaCO_3$ (1.2 g, 12 mmol) in 4 mL of H_2O and DCM (4 mL)) during 2 h at r.t. The reaction mixture was extracted by DCM (3 × 10 mL), washed with brine (5 mL) and dried over anhydrous $MgSO_4$. Final ITC **1** was isolated after evaporation of solvent in good yield (53 %) (**Figure S1**) [1]. Known compound.

*Ethyl isothiocyanate (**1**).* Colorless oil. Yield 53 % (0.092 g, 1.06 mmol). ^1H NMR (400 MHz, $CDCl_3$): δ 3.58 (*q*, *J*= 7.1 Hz, 2 H, C*H*$_2$), 1.40 (*t*, *J*= 7.1 Hz, 3 H, C*H*$_3$). EI-MS: *m/z* $[M]^+$ calcd for $C_3H_5NS^+$ 87.14, found 87.00. The analytical data are in agreement with those reported previously in the literature [2].

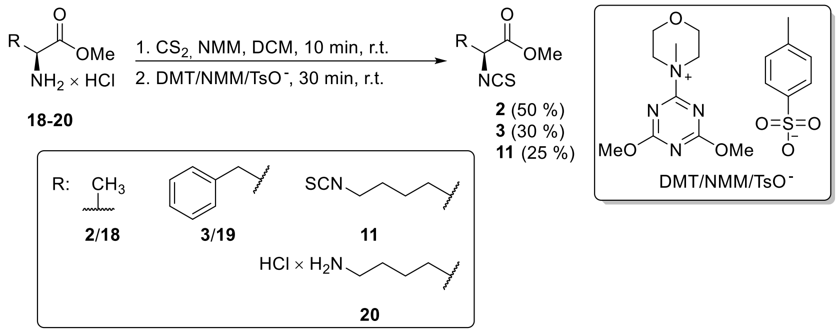

Figure S2. Synthesis of isothiocyanate derivatives of amino acids methyl esters **2**, **3** and **11** using DMT/NMM/TsO⁻ as desulfurating reagent.

Isothiocyanate derivatives of amino acids methyl esters **2**, **3** and **11** were synthesized in one-pot, two step procedure using 4-(4,6-dimethoxy-1,3,5-triazin-2-yl)-4-methylmorpholinium toluene-4-sulfonate (DMT/NMM/TsO⁻) as a desulfurating agent. In the first step hydrochloride **18-20** (2 mmol), carbon disulfide (CS_2) (0.36 mL, 6 mmol for **18-19** or 0.72 mL, 12 mmol for **20**) and *N*-methylmorpholine (NMM) (0.66 mL, 6 mmol for **18-19** or 1.32 mL, 12 mmol for **20)** were stirred in DCM (5 mL) at r.t. for 10 min, after which DMT/NMM/TsO⁻ (0.828 g, 2 mmol for **18-19** or 1.656 g, 4 mmol for **20**) was added and stirring was continued for 30 min at r.t. Next, the reaction mixture was diluted with DCM (50 mL) and washed with H_2O (5 mL), 1 N HCl (2 × 5 mL), H_2O (5 mL), and dried over anhydrous $MgSO_4$. Final isothiocyanates **2**, **3** and **11** were isolated after flash chromatography (hexane: EtOAc 20: 1) in low yields (25-50 %) (**Figure S2**) [3]. Known compounds.

*(S)-Methyl 2-Isothiocyanatopropanoate (**2**).* Colorless oil. Yield 50 % (0.145 g, 1 mmol). ^{1}H NMR (700 MHz, $CDCl_3$): δ = 4.35 (*q*, *J*= 7.1 Hz, 1 H, C*H*NCS), 3.81 (*s*, 3 H, C*H*3O), 1.60 (*d*, *J*= 7.1 Hz, 3 H, C*H*3). HR-MS: *m/z* $[M]^+$ calcd for $C_5H_7NO_2S^+$ 145.0197, found 145.0204.

*(S)-Methyl 2-isothiocyanato-3-phenylpropanoate (**3**).* Colorless oil. Yield 30 % (0.132 g, 0.6 mmol). ^{1}H NMR (700 MHz, $CDCl_3$): δ = 7.36–7.34 (*m*, 2 H, CH_{Ar}), 7.31–7.29 (*m*, 1 H, CH_{Ar}), 7.23–7.22 (*m*, 2 H, CH_{Ar}), 4.48 (*dd*, $J_{H\alpha H\beta}$ = 8.4 Hz, $J_{H\alpha H\gamma}$ = 4.8 Hz, 1 H, CH_αNCS), 3.80 (*s*, 3 H, CH_3O), 3.25 (*dd*, $J_{H\gamma H\beta}$ = 13.8 Hz, $J_{H\gamma H\alpha}$ = 4.7 Hz, 1 H, CH_γPh), 3.13 (*dd*, $J_{H\beta H\gamma}$ = 13.8 Hz, $J_{H\beta H\alpha}$ = 8.4 Hz, 1 H, CH_βPh). HRMS: *m/z* $[M]^+$ calcd for $C_{11}H_{11}NO_2S^+$ 211.0510, found 221.0506.

*(S)-Methyl 2,6-diisothiocyanatohexanoate (**11**).* Colorless oil. Yield 25 % (0.122 g, 0.5 mmol). ^{1}H NMR (700 MHz, $CDCl_3$): δ = 4.32 (2 × *d*, *J* = 7.8 Hz, *J* = 7.8 Hz, 1 H, C*H*NCS), 3.83 (*s*, 3 H, CH_3O), 3.56 (*t*, *J* = 6.5 Hz, 2 H, CH_2NCS), 1.99–1.91 (*m*, 2 H, CH_2), 1.78–1.73 (*m*, 2 H, CH_2), 1.61–1.57 (*m*, 2 H, CH_2). HRMS: *m/z* $[M]^+$ calcd for $C_9H_{12}N_2O_2S_2^+$ 244.0343 found 244.0340.

The analytical data of ITCs **2**, **3** and **11** are in agreement with those reported previously in the literature [3].

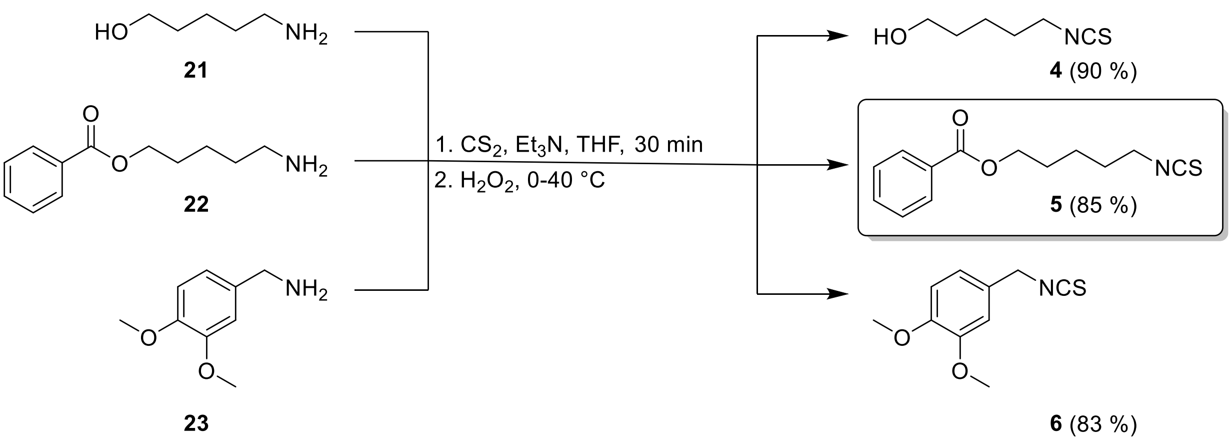

Figure S3. Synthesis of ITC **4–6** using H_2O_2 as desulfurating reagent.

In a round-bottom flask equipped with magnetic bar primary amines **21-23** (10 mmol), *N*,*N*-diisopropylethylamine (10 mmol) was dissolved in THF (10 mL) and the solution was cooled in an ice bath to 0–10 °C. Next, carbon disulfide (50 mmol) was added and the reaction mixture was stirred for 30 min. After this time, 30 % H_2O_2 (50 mmol) was added dropwise with temperature control and continued stirring. After 15 min, the reaction mixture was acidified with 2 M HCl to pH 1–2 and THF was evaporated under reduce pressure. The solid residue was extracted with EtOAc (3 × 50 mL), and the organic layer was dried over anhydrous $MgSO_4$. Pure ITCs **4-6** were isolated after flash chromatography (hexane : EtOAc 5 : 1) (**Figure S3**) [4].

*5-Isothiocyanatopentanol (**4**).* Colorless oil. Yield 90 % (1.3 g, 9 mmol). ^{1}H NMR (400 MHz, $CDCl_3$): δ 3.67 (*t*, *J*= 6.2 Hz, 2 H, CH_2OH), 3.55 (*t*, *J* = 6.5 Hz, 2 H, CH_2NCS), 1.79–1.68 (*m*, 3 H, CH_2CH$_2$O*H*), 1.65–1.45 (*m*, 4 H, CH_2CH_2CH$_2$NCS). HRMS: *m/z* $(M+H)^+$ calcd for $C_6H_{12}NOS^+$ 146.0640 found 146.0777. The analytical data are in agreement with those reported previously in the literature [5]

5-(Benzoyloxy)pentyl isothiocyanate (**5**). Colorless oil. Yield 85 %. ^{1}H NMR (300 MHz, $CDCl_3$); δ 8.07–8.02 (*m*, 2H, Ar-*H*), 7.59–7.53 (*m*, 1H, Ar-*H*), 7.48–7.41 (*m*, 2H, Ar-*H*), 4.34 (*t*, *J* = 6.4 Hz, 2H, OCH_2), 3.55 (*t*, *J* = 6.4 Hz, 2H, CH_2NCS), 1.88–1.73 (*m*, 4H, 2 × CH_2), 1.66–1.54 (*m*, 2H, CH_2). HRMS: *m/z* $[M+H]^+$ calcd for $C_{13}H_{16}NO_2S^+$ 250.0896, found 250.0890. New compound.

*3,4-Dimethoxybenzyl isothiocyanate (**6**).* Colorless oil. Yield 83 % (1.73 g, 8.3 mmol). ^{1}H NMR (400 MHz, $CDCl_3$): δ 7.30–6.74 (*m*, 3 H, CH_{Ar}), 4.63 (*s*, 2 H, CH_2), 3.90 (*s*, 3 H, CH_3), 3.88 (*s*, 3 H, CH_3). HRMS: *m/z* $(M+H)^+$ calcd for $C_{10}H_{12}NO_2S^+$ 210.0589 found 210.0611. The analytical data are in agreement with those reported previously in the literature [6].

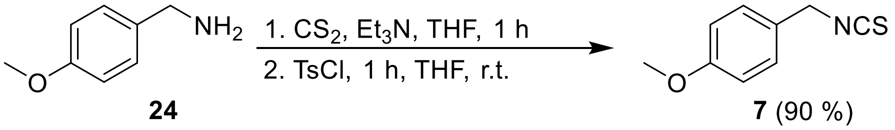

Figure S4. Synthesis of ITC **7** using TsCl as desulfurating agent.

Isothiocyanate **7** was also prepared in THF (10 mL), in one-pot, two-step procedure using primary amine **24** (1.1 mmol) as a substrate and in the present of triethylamine (0.5 mL, 3.6 mmol) and CS_2 (0.06 mL, 1.1 mmol). In this reaction tosyl chloride (0.23 g, 1.2 mmol) was used as desulfurating reagent. Product **7** was also isolated in high yield (90 %) after flash chromatography (hexane 100%) (**Figure S4**) [7]. Known compound.

*4-Methoxybenzyl isothiocyanates (**7**).* Colorless oil. Yield 90 % (0.177 g, 1 mmol). ^{1}H NMR (400 MHz, $CDCl_3$): δ 7.24-7.23 (*m*, 2 H, CH_{Ar}), 6.92-6.89 (*m*, 2 H, CH_{Ar}), 4.63 (*s*, 2 H, CH_2), 3.81 (*s*, 3 H, OCH_3). HRMS: *m/z* $(M-H)^-$ calcd for C_9H_8NOS 178.0332, found: 178.0327.The analytical data are in agreement with those reported previously in the literature [8].

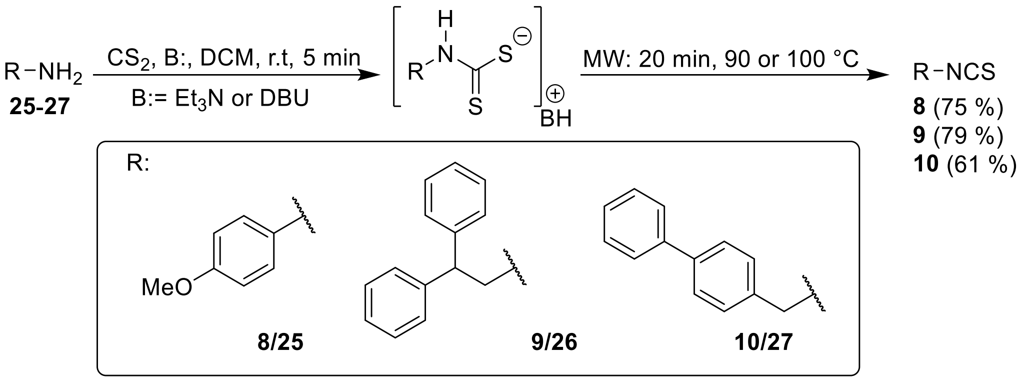

Figure S5. Microwave-assisted synthesis of isothiocyanates **8**–**10**.

Isothiocyanates **8**–**10** were obtained in one pot, two step microwave-assisted (MW) synthesis, without using additional desulfurating agent. Aliphatic primary amines **26**–**27** (2 mmol) or aromatic amine **25** (2 mmol) in the presence of carbon disulfide (CS_2) (0.36 mL, 6 mmol) and triethylamine (Et_3N) (for amines **26**–**27**, 1.11 mL, 8 mmol) or DBU (for amine **25**, 1.2 mL, 8 mmol) were transformed in standard reaction in dry DCM (2 mL) at r.t. into intermediate dithiocarbamates. Next, intermediate dithiocarbamates were converted to ITCs **8**–**10** in microwave reactor (20 min, 90 °C for **26**–**27** or 20 min, 100 °C for **25**). Final products were isolated after purification using flash chromatography (hexane 100%) in high yields (61–79 %) (**Figure S5**) [9]. Known compounds.

*1-Isothiocyanato-4-methoxybenzene (**8**).* Colorless oil. Yield 75 % (0.247 g, 1.5 mmol). ^{1}H NMR (700 MHz, $CDCl_3$): δ = 7.16 (*d*, *J*= 9.1 Hz, 2 H, CH_{Ar}), 6.85 (*d*, *J*= 9.1 Hz, 2 H, CH_{Ar}), 3.81 (s, 3 H, OCH_3). HRMS: *m/z* $(M+H)^+$ calcd for $C_8H_8NOS^+$ 166.0321 found 166.0318. The analytical data are in agreement with those reported previously in the literature [9].

*(2-Isothiocyanatoethane-1,1-diyl)dibenzene (**9**).* Colorless oil. Yield 79 % (0.378 g, 1.58 mmol). ^{1}H NMR (700 MHz, $CDCl_3$): δ = 7.37–7.34 (*m*, 4 H, CH_{Ar}), 7.29–7.27 (*m*, 2 H, CH_{Ar}), 7.25–7.23 (*m*, 4 H, CH_{Ar}), 4.38 (*t*, *J*= 7.5 Hz, 1 H, C*H*), 4.09 (*d*, *J*= 7.5 Hz, 2 H, CH_2NCS). HRMS: *m/z* $(M+H)^+$ calcd for

$C_{15}H_{14}NS^+$ 239.08 found 240.10. The analytical data are in agreement with those reported previously in the literature [9].

*4-(Isothiocyanatomethyl)-1,1'-biphenyl (**10**).* White solid, mp 62–64 °C (lit.[9] 63–64 °C). Yield 61 % (0.274, 1.22 mmol). ^{1}H NMR (700 MHz, $CDCl_3$): δ = 7.63–7.58 (*m*, 4 H, C*H*$_{Ar}$), 7.47–7.44 (*m*, 2 H, C*H*$_{Ar}$), 7.40–7.36 (*m*, 3 H, C*H*$_{Ar}$), 4.76 (*s*, 2 H, C*H*$_2$). HRMS: *m/z* $(M)^+$ calcd for $C_{14}H_{11}NS^+$ 225.0612 found 225.0613. The analytical data are in agreement with those reported previously in the literature [9].

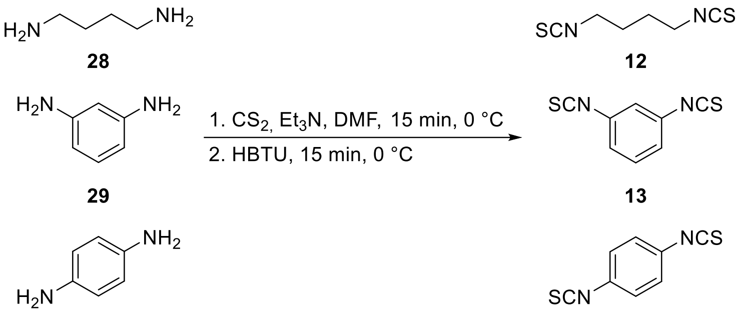

Figure S6. Synthesis of diisothiocyanates **12−14** using HBTU as desulfurating agent.

Diisothiocyanates **12−14** were synthesized in one-pot, two step procedure, wherein diamines **28−30** (10 mmol), CS_2 (200 mmol) and Et_3N (20 mmol) were dissolved in DMF (20 mL) at 0 °C and stirred for 15 min. Subsequently, HBTU (15 mmol) was added and the reaction mixture was stirred at 0 °C for additional 15 min. Products were isolated after column chromatography using eluent EtOAc : hexane 1:1 (**Figure S6**) [10].

*1,4-Diisothiocyanatobutane (**12**).* White solid, mp 125 °C (lit.[11] 124 °C). Yield 50 % (0.865 g). ^{1}H NMR (700 MHz, $CDCl_3$): δ = 3.71 (*t, J*= 7.1 Hz, 4 H, 2 × C*H*$_2$NCS), 1.80 (*m*, 4 H, 2 × C*H*$_2$). HRMS: *m/z* $(M+H)^+$ calcd for $C_6H_9N_2S_2^+$ 173.0202 found 173.0200. The analytical data are in agreement with those reported previously in the literature [11].

*1,3-Diisothiocyanatobenzene (**13**).* White solid, mp 52–54 °C (lit.[9] 50–51 °C). Yield 66 % (1.267 g). ^{1}H NMR (700 MHz, $CDCl_3$): δ = 7.33 (*t, J*= 8.3 Hz, 1 H, C*H*$_{Ar}$), 7.12 (*dd, J*= 8.1 Hz, *J*= 2.0 Hz, 2 H, C*H*$_{Ar}$), 7.05 (*t, J*= 2.0 Hz, 1 H, C*H*$_{Ar}$). HRMS: *m/z* $(M+H)^+$ calcd for $C_8H_5N_2S_2^+$ 192.9889 found 192.9885. The analytical data are in agreement with those reported previously in the literature [9].

*1,4-Disothiocyanatobenzene (**14**).* Yield 80 % (1.53 g). White solid, mp 130–131 °C (lit.[12] 132–133 °C). ^{1}H NMR (700 MHz, $CDCl_3$) δ 7.21 (s, 4 H). HRMS: *m/z* $(M+H)^+$ calcd for $C_8H_5N_2S_2^+$ 192.9889 found 192.9891. The analytical data are in agreement with those reported previously in the literature [12].

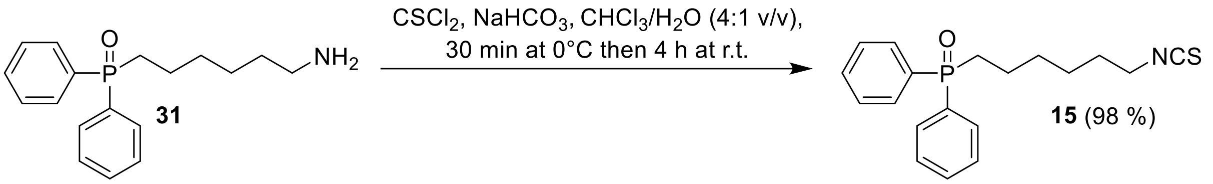

Figure S7. Synthesis 6-(isothiocyanatohexyl)diphenylphosphino oxide (**15**) using thiophosgene.

6-(Isothiocyanatohexyl)diphenylphosphino oxide (**15**) was synthesized using amine **31** (0.3 mmol) reaction with thiophosgene ($CSCl_2$) (0.03 mL, 0.375 mmol) and $NaHCO_3$ (0.1 g, 1.2 mmol), under two-phase chloroform-aqueous 1 M NaOH (8 mL : 2 mL) system. The reaction was stirred for 30 min at 0 °C and 4 h at r.t. Next, $CHCl_3$ (50 mL) was added and phases were separated. Organic phase was washed with H_2O (5 mL), 1 N HCl (5 mL), H_2O (5 mL) and brine (5 mL) and dried over anhydrous $MgSO_4$. Product was isolated in almost quantitative yield (98 %) after flash chromatography (hexane : acetone 2 : 1). (**Figure S7**) [13]. Known compound.

*(6-Isothiocyanatohexyl)diphenylphosphine oxide (**15**).* Colorless oil. Yield 98 % (0.1 g, 0.29 mmol). ^{31}P NMR (283 MHz, $CDCl_3$): 32.12. ^1H NMR (700 MHz, $CDCl_3$): δ 7.75–7.72 (4 H, *m*, CH_{Ar}), 7.53–7.50 (2 H, *m*, CH_{Ar}), 7.48–7.46 (4 H, *m*, CH_{Ar}), 3.46 (2 H, *t*, *J*= 6.6 Hz, $C_{(6)}H_2NCS$), 2.28–2.24 (2 H, *m*, CH_2), 1.67–1.62 (4 H, *m*, 2 × CH_2), 1.46–1.37 (4 H, *m*, 2 × CH_2). ESI–MS: *m/z* $[M+H]^+$ calced for $C_{19}H_{23}NOPS^+$ 344.1232, found 344.1242. The analytical data are in agreement with those reported previously in the literature [13].

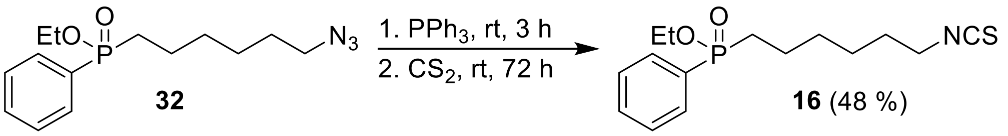

Figure S8. Synthesis of ITC **16** using Staudinger/aza-Wittig reaction.

Ethyl (6-isothiocyanatohexyl)(phenyl)phosphinate (**16**) was obtained in tandem Staudinger/aza-Wittig. Azide **32** (1 mmol) in reaction with triphenylphosphine (Ph_3P) (0.289 g, 1.1 mmol) and CS_2 (0.48 mL, 8 mmol) was transformed in toluene (5 mL) in good yield (48 %) to final product **16** after flash chromatography (hexane : acetone 3:1) (**Figure S8**) [13]. Known compound.

*Ethyl (6-isothiocyanatohexyl)(phenyl)phosphinate (**16**).* Colorless oil. Yield 48 % (0.149 g, 0.48 mmol). ^{31}P NMR (283 MHz, $CDCl_3$): δ 44.36. ^1H NMR (700 MHz, $CDCl_3$): δ 7.75–7.72 (2 H, *m*, CH_{Ar}), 7.53–7.51 (1 H, *m*, CH_{Ar}), 7.45 (2 H, *td*, J_{HH}= 7.4 Hz, J_{HP}= 3.2 Hz, CH_{Ar}), 4.07–4.01 (1 H, *m*, H_α from CH_2O), 3.83–3.78 (1 H, *m*, H_β from CH_2O), 3.43 (2 H, *t*, J_{HH}= 6.6 Hz, $C_{(6)}H_2NCS$), 1.93–1.86 (1 H, *m*, H_α from CH_2), 1.84–1.78 (1 H, *m*, H_β from CH_2), 1.63– 1.45 (4 H, *m*, 2 × CH_2), 1.37–1.33 (4 H, *m*, 2 × CH_2), 1.25 (3 H, *t*, J_{HH}= 7.0 Hz, CH_3). EI–HRMS: *m/z* $[M]^{+\cdot}$ calced. for $C_{15}H_{22}NO_2PS^{+\cdot}$ 311.1109, found 311.1107. The analytical data are in agreement with those reported previously in the literature [13].

BITC and **PEITC** were synthesized, in yield 82 % and 87 % yields, respectively, according to the procedure shown in **Figure S5** [7] in the presence of Et_3N. **AITC** has been synthesized in one-pot, two step procedure using propane phosphonic acid anhydride (T3P®) as desulfurating agent [14]. **SFN** was commercially available (Sigma-Aldrich, Poland)

Table S1. Molecular docking scores obtained for best models of all tested compounds, divided based on binding cavity occupied by the ligand, and visualization of docking pose for best scoring models.

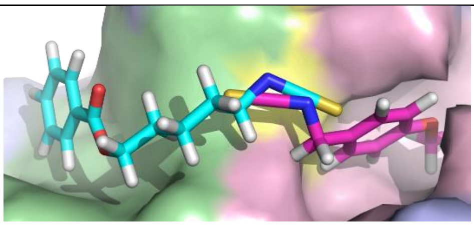

Compound	Docking scores (large cavity)	Docking scores (small cavity)	Best scoring docking model
BITC		-55.5377	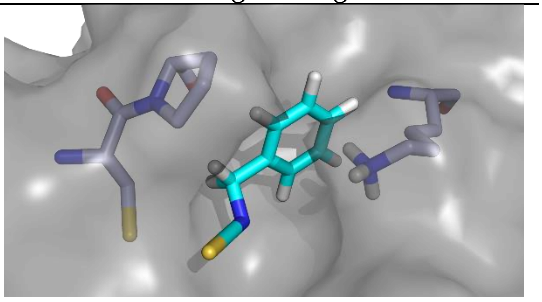
PEITC		-59.3983	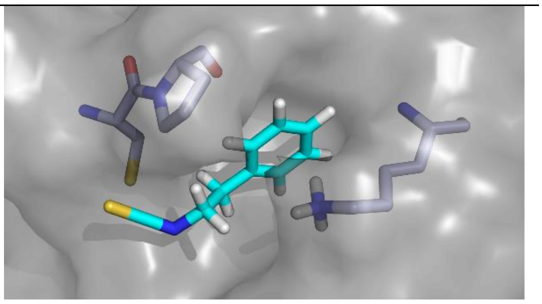
SFN		-50.1264	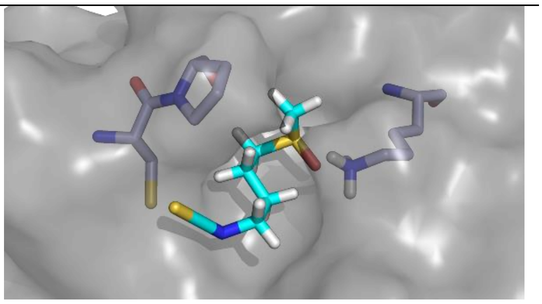
AITC		-60.6461	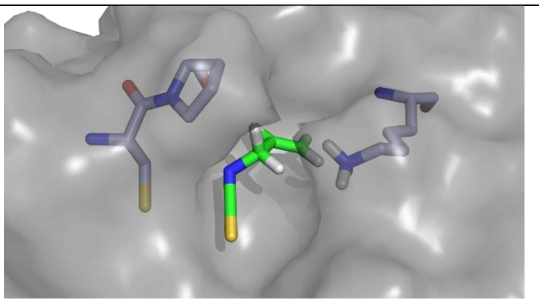

1		-45.472	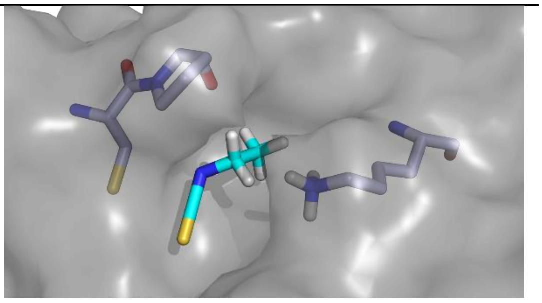
2		-56.5898	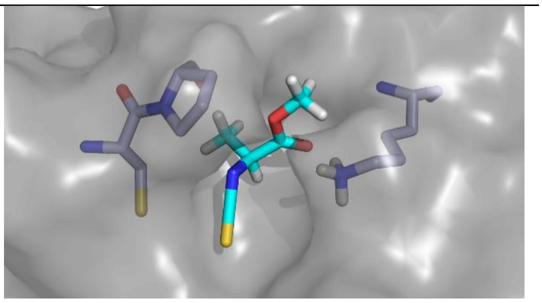
3		-64.9994	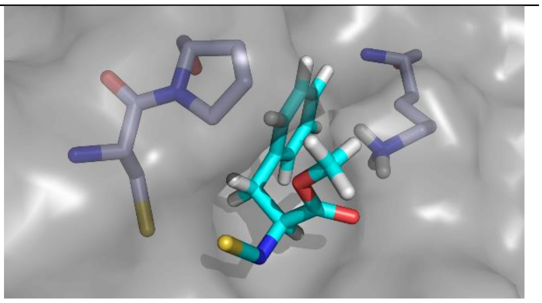
4		-63.6047	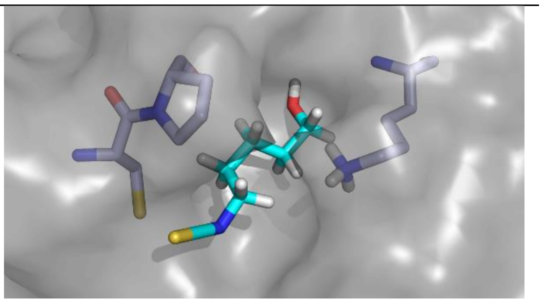
5	-77.618		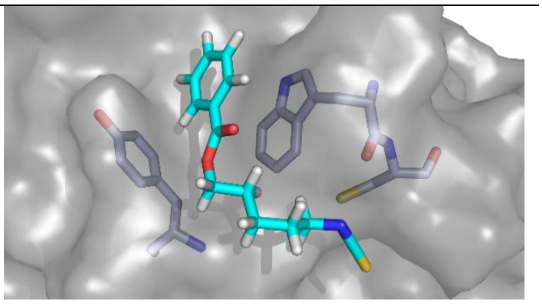

6		-62.8034	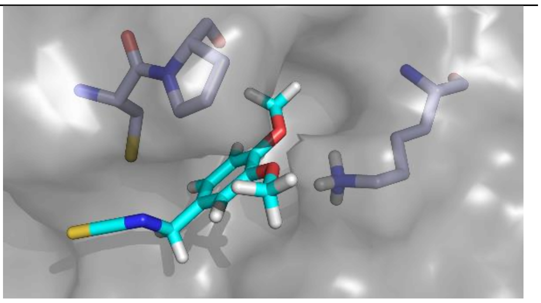
7		-59.9651	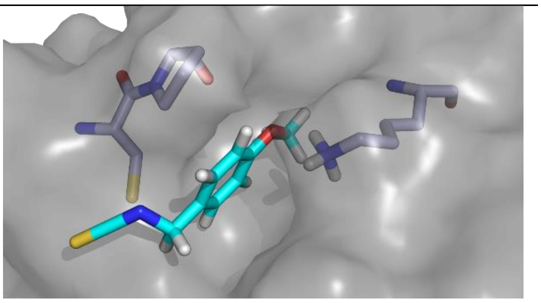
8		-55.1661	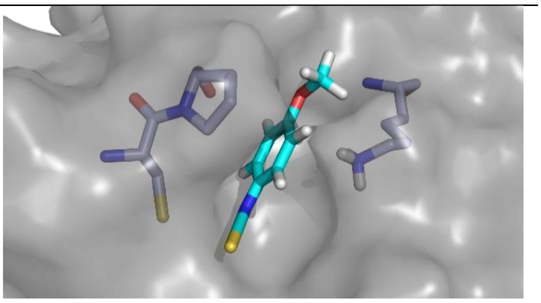
9	-65.6743		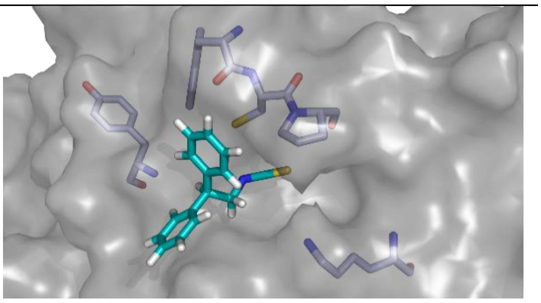
10	-70.3154		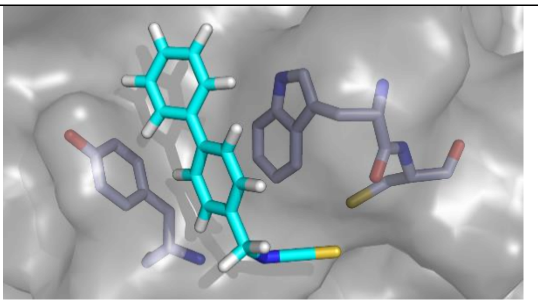

11		-68.4147	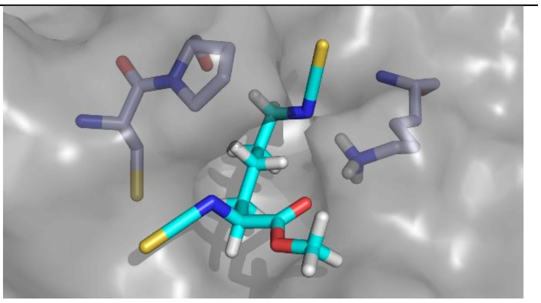
12		-60.8435	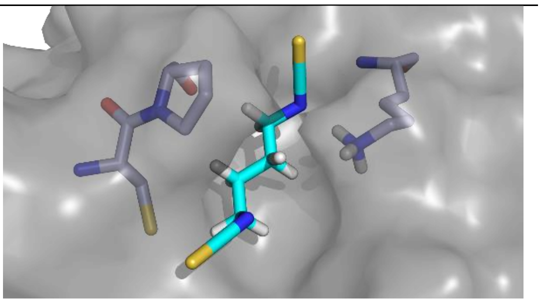
13		-54.9529	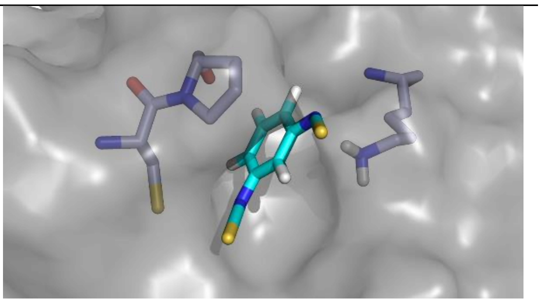
14		-56.5582	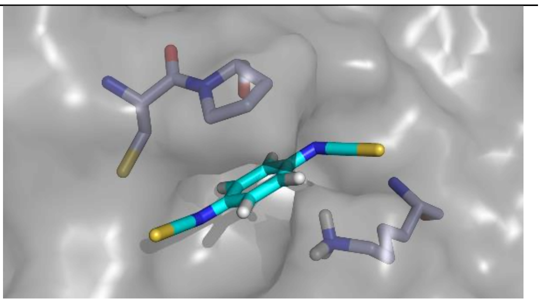
15	-73.1809		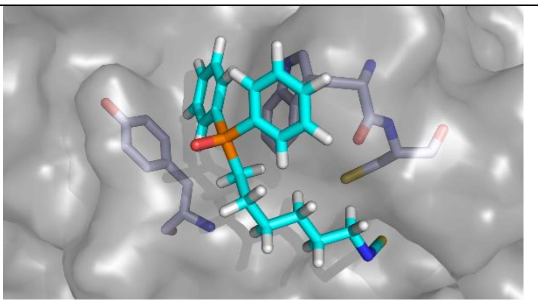

16	-77.7917	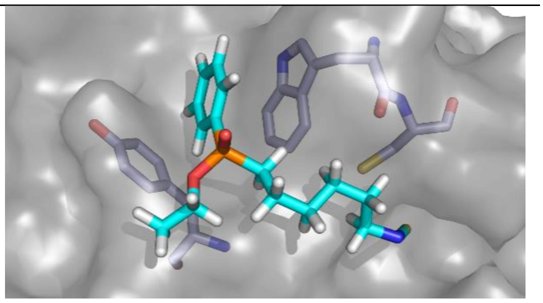

3. References

[1]. Nam, K. D.; Han, M.; Yoon, J.; Kim, E.-A.; Cho, S.-W.; Hahn, H.-G. 2-Amino-1,3-thiazoles Suppressed Lipopolysaccharide-Induced IL-β and TNF-α. *Bull. Korean Chem. Soc.* **2013**, *34*, 271

[2]. Li, D.; Shu, Y.; Li, P.; Zhang, W.; Ni, H.; Cao, Y. Synthesis and structure-activity relationships of aliphatic isothiocyanate analogs as antibiotic agents. *Med. Chem. Res.* **2013**, *22*, 3119–3125.

[3]. Janczewski, Ł.; Kręgiel, D.; Kolesińska, B. Synthesis of Isothiocyanates Using DMT/NMM/TsO− as a New Desulfurization Reagent. *Molecules* **2021**, *26*, 2740.

[4]. Li, G.; Tajima, H.; Ohtani, T. An Improved Procedure for the Preparation of Isothiocyanates from Primary Amines by Using Hydrogen Peroxide as the Dehydrosulfurization Reagent. *J. Org. Chem.* **1997**, *62*, 4539-4540.

[5]. Park, S.; Hayes, B. L.; Marankan, F.; Mulhearn, D. C.; Wanna, L.; Mesecar, A. D.; Santarsiero, B. D.; Johnson, M. E.; Venton, D. L. Regioselective covalent modyfication of hemoglobin in search of antisicking agents. *J. Med. Chem.* **2003**, *46*, 936-953.

[6]. De Nicola, G, R.; Montaut, S.; Rollin, P.; Nyegue, M.; Menut, C.; Iori, R.; Tatibouët, A. Stability of benzylic-type isothiocyanates in hydrodistillation-mimicking conditions. *J. Agric. Food Chem.* **2013**, *61*, 137-142.

[7]. Wong, R.; Dolmann, S. J. Isothiocyanates from Tosyl Chloride Mediated Decomposition of in Situ Generated Dithiocarbamic Acid Salts. *J. Org. Chem*. **2007**, *72*, 3969-3971.

[8]. Kiaku, C.; Walsh, Leech, M. C.; Poole, D. L.; Mason, J.; Goodall, I. C. A.; Devo, P.; Lam, K. Eletrochemical isothiocyanation of primary amines. *Org. Lett.* **2023**, *25*, 1147-1150.

[9]. Janczewski, Ł.; Gajda, A.; Gajda, T. Direct, Microwave-Assisted Synthesis of Isothiocyanates. *Eur. J. Org. Chem.* **2019**, *2019*, 2528–2532.

[10]. Grzywa, R.; Winiarski, Ł.; Psurski, M.; Rudnicka, A.; Wietrzyk, J.; Gajda, T.; Oleksyszyn, J. Synthesis and biological activity of diisothiocyanate-derived mercapturic acids. *Bioorg. Med. Chem. Lett.* **2016**, *26*, 667–671.

[11]. Tournaire-Arellano, C.; Younes-El Hage, S.; Valès, P.; Caujolle, R.; Sanon, A.; Bories, C.; Loiseau, P. M. Synthesis and biological evaluation of ureido and thioureido derivatives of 2-amino-2-deoxy-d-glucose and related aminoalcohols as *N*-acetyl-β-d-hexosaminidase inhibitors. *Carbohydr. Res.* **1998**, *314*, 47-63.

[12]. Liao, Y. –Y.; Deng, J. –C.; Ke, Y. –P.; Zhong, X. –L.; Xu, L.; Tang, R. –Y.; Zheng, W. Isothiocyanation of amines using the Langlois reagent. *Chem. Commun.* **2017**, *53*, 6073-6076.

[13]. Janczewski, Ł.; Psurski, M.; Świtalska, M.; Gajda, A.; Goszczyński, T.M.; Oleksyszyn, J.; Wietrzyk, J.; Gajda, T. Design, Synthesis, and Evaluation of ω-(Isothiocyanato)alkylphosphinates and Phosphine Oxides as Antiproliferative Agents. *ChemMedChem* **2018**, *13*, 105–115.

[14]. Janczewski, Ł.; Gajda, A.; Frankowski, S.; Goszczyński, T.M.; Gajda, T. T3P® – A Benign Desulfurating Reagent in the Synthesis of Isothiocyanates. *Synthesis (Stuttg).* **2018**, *50*, 1141–1151.
